# Physiological and agronomical traits effects of titanium dioxide nanoparticles in seedlings of *Solanum lycopersicum* L

**DOI:** 10.1186/s12870-024-04763-9

**Published:** 2024-02-28

**Authors:** Ricardo Tighe-Neira, Marjorie Reyes-Díaz, Adriano Nunes-Nesi, Jaciara Lana-Costa, Gonzalo Recio, Erico R. Carmona, Patricio Acevedo, Zed Rengel, Claudio Inostroza-Blancheteau

**Affiliations:** 1 Programa de Doctorado en Ciencias Agropecuarias, Facultad de Recursos Naturales, Universidad Católica de Temuco, P.O. Box 15-D, Temuco, Chile; 2 Laboratorio de Fisiología y Biotecnología Vegetal, Departamento de Ciencias Agropecuarias y Acuícolas, Facultad de Recursos Naturales, Universidad Católica de Temuco, P.O. Box 15-D, Temuco, Chile; 3 Departamento de Ciencias Químicas y Recursos Naturales, Facultad de Ingeniería y Ciencias, Universidad de La Frontera, P.O. Box 54-D, Temuco, Chile; 4 Center of Plant, Soil Interaction and Natural Resources Biotechnology, Scientific and Technological Bioresource Nucleus (BIOREN), Universidad de La Frontera, P.O. Box 54-D, Temuco, Chile; 5 Departamento de Biologia Vegetal, Universidade Federal de Viçosa, Viçosa, 36570-900 Minas Gerais Brazil; 6 Facultad de Ingeniería, Arquitectura y Diseño, Universidad San Sebastián, Lientur 1457, Concepción, Chile; 7 Laboratorio de Bio-nanomateriales, Facultad de Recursos Naturales Renovables, Universidad Arturo Prat, Av. Arturo Prat s/n Campus Huayquique, Iquique, Chile; 8 Departamento de Ciencias Físicas, Facultad de Ingeniería y Ciencias, Universidad de La Frontera, Temuco, Chile; 9 Center for Optics and Photonics, Universidad de Concepcion, Casilla, Concepción, 4012 Chile; 10 Soil Science and Plant Nutrition, UWA School of Agriculture and Environment, The University of Western Australia, Perth, WA Australia; 11 Institute for Adriatic Crops and Karst Reclamation, Split, Croatia; 12 Núcleo de Investigación en Producción Alimentaria, Facultad de Recursos Naturales, Universidad Católica de Temuco, P.O. Box 15-D, Temuco, Chile

**Keywords:** Soluble sugars, Starch, Protein, Biomass, Photosynthesis

## Abstract

**Background:**

Titanium dioxide nanoparticles (TiO_2_ NPs) have been reported to have contrasting effects on plant physiology, while their effects on sugar, protein, and amino acid metabolism are poorly understood. In this work, we evaluated the effects of TiO_2_ NPs on physiological and agronomical traits of tomato (*Solanum lycopersicum* L.) seedlings. Tomato seeds were treated with TiO_2_ NPs (1000 and 2000 mg L^− 1^), TiO_2_ microparticles (µPs, 2000 mg L^− 1^) as the size control, and ultrapure water as negative control.

**Results:**

The dry matter of stems (DMs), leaves (DMl) and total dry matter (DMt) decreased as particle concentration increased. This trend was also observed in the maximum quantum yield of light-adapted photosystem II (PSII) (F_v_´/F_m_´), the effective quantum yield of PSII (ΦPSII), and net photosynthesis (P_n_). The concentrations of sugars, total soluble proteins, and total free amino acids were unaffected, but there were differences in the daily dynamics of these compounds among the treatments.

**Conclusion:**

Our results suggest that treating tomato seeds with TiO_2_ might affect PSII performance, net photosynthesis and decrease biomass production, associated with a concentration- and size-related effect of TiO_2_ particles.

**Supplementary Information:**

The online version contains supplementary material available at 10.1186/s12870-024-04763-9.

## Background

Since the end of the 20th century, nanotechnology has been considered the most useful tool for tackling various health, energy, and environmental challenges [1]. Nanomaterials with an average size of < 100 nm are commonly named nanoparticles (NPs), and they are used, in agriculture as nanofertilizers, soil amendments, soil conditioners, pesticides, and plant growth promoters [2–4]. Titanium dioxide is the most widely-used compound in the manufacture of nanoparticles (TiO_2_ NPs), with a production of over 2 million tons per year [5, 6]. There are two crystalline structures of TiO_2_ NPs, anatase and rutile, of which anatase is considered to have a greater impact on biological organisms [7]. Most studies utilize priming as a means of exposure, as it is seen as an innovative, sustainable and practical agricultural strategy that has significant impacts on growth, physiological and biochemical aspects of plants under normal and stress conditions [8–10]. TiO_2_ NPs could have positive effects (e.g. increase in biomass production and resistance to specific abiotic stresses, biostimulation) and negative effects on plants (e.g. inhibition of plant growth and/or development) [11], with contrasting effects reported on crops at the physiological, metabolic, and productivity levels, as the NP-plant interaction is species-specific. Regarding productivity, TiO_2_ NPs decreased biomass in *Ocimum basilicum* L [12]. , *Oryza sativa* [13], *Zea mays* L [14]. , and *Solanum lycopersicum* [15]. However, other authors have reported increases in biomass production of *S. lycopersicum* [16] and *Glycine max* L [17]. , with a neutral effect on biomass reported in *Triticum aestivum* and *Brassica napus* L [5]. . Regarding the structure and functioning of the photosynthetic machinery, there are reports that TiO_2_ NPs cause a decrease in chlorophyll content in *T. aestivum* at concentrations ranging from 10 to 40 mg L^− 1^ [18]. In the same line, [19] found a decrease in CO_2_ fixation, transpiration, and stomatal conductance in *O. sativa* L., but no effect on photochemical parameters was observed at a concentration of 1000 mg kg^− 1^. In addition, recent reports using *S. lycopersicum* indicate that concentrations of TiO_2_ NPs lower than 2000 mg L^− 1^ applied to roots had positive effects on photosynthetic activity, while concentrations higher than 2000 mg L^− 1^ had a negative impact [20]. . However, in the same species, negative impacts on photosynthesis were observed using concentrations of 5 to 160 mg L^− 1^ [21]. Regarding metabolic changes, more than 70% of metabolites in *T. aestivum* L [22]. changed in a concentration-dependent manner after TiO_2_ NP application. Similar results were found in *O. sativa* L [13]. , with 105 metabolites accumulated differentially between the control and TiO_2_ NP-treated plants. In addition, the same authors observed an inhibition of carbohydrate synthesis and an increase in amino acid and secondary metabolites; however, the mechanisms behind the metabolic impacts of TiO_2_ NPs are still unclear [22].

*S. lycopersicum* is an important crop grown worldwide [20]. This plant species has been used in the evaluation of the toxicity, absorption, transport, and accumulation of TiO_2_ NPs applied in concentrations ranging from 0 to 5000 mg L^− 1^ [23]. In addition, the effects of TiO_2_ NPs on germination, growth, biomass production, photosynthesis, water conductance, transpiration, and antioxidant systems have been evaluated [24–26]. These studies [23–26] invariably apply the NPs at the seedling or adult plant stage. Nevertheless, there is little knowledge on the daily dynamics of gas exchange parameters, sugar and protein biosynthesis, and biomass production when seeds of *S. lycopersicum* are primed with TiO_2_ NPs. Seed priming has multiple advantages, such as the lower volume of NPs required to treat a far greater number of plants, dose consistency among plants, and lack of possible detrimental effects on unintended targets as the treatment is not performed in the field. Thus, the aim of the present work was to evaluate the effect of TiO_2_ NPs applied to seeds on physiological and agronomical traits of *S. lycopersicum* seedlings.

## Materials and methods

### Electromagnetic spectrum and physical characterization of NPs

A spectroradiometer (Licor, 1800, Lincoln, NE, USA) was used to confirm the electromagnetic spectrum inside the growth chamber. This is relevant since TiO_2_ NPs are activated by UV-A. The NPs used in this research (< 100 nm, catalog #637,262, Sigma Aldrich Co., St. Louis, MO, USA) were the same as those employed in a previous study [21], characterized according to Nanogenotox [27].

### Plant material, treatments and growth conditions

*S. lycopersicum* seeds (cv. Cal-Ace) were treated with TiO_2_ nanoparticles (NPs) (1000 and 2000 mg L^− 1^) and TiO_2_ microparticles (µPs, 2000 mg L^− 1^) as the size control. Ultrapure water was used as negative control. A size control is necessary to assess ‘particle agglomeration’, which is high for NPs, but very low for µPs. These NP and µP concentrations were adopted based on a previous study [20]. The TiO_2_ NPs and µPs (rutile) were suspended in ultrapure water and stirred for 30 min, followed by a further 30 min in a sonicator (Elmasonic VC300) at room temperature. The treatments were applied by seed imbibition in a 10 mL volume in a Petri dish for 72 h, according to [28]. Afterwards, six germinated seeds of *S. lycopersicum* were transferred to individual pots (500 mL) containing a substrate (peat + perlite, volumetric ratio 2:1). Six replicate pots, each with one germinated seed, were placed in a large box (3 L) for irrigation with distilled water by capillarity.

Plants were grown in a controlled-environment chamber under 200 µmol photons m^− 2^ s^− 1^, 23 ± 1 ºC, 50% relative humidity, and a 16/8 h photoperiod for 30 days. The growth chamber was equipped with UV-A lamps (UV-A Hanging, 40 W, 48 inches; 315–400 nm, at a light intensity of 6.9 µmol photons m^− 2^ s^− 1^) and standard photosynthetic active radiation (PAR) LEDs (400–800 nm).

### Determination of biomass production

After 30 days of exposure to the different treatments, plants were separated into leaves, stems, and roots, and each portion weighed after drying in a forced-air oven at 60 °C to constant weight. Dry matter (DM) was calculated according to [29].

### Water loss measurement using excised leaves

Water loss was determined using mature and fully-expanded leaves, which were excised as described previously [30]. Weight loss was recorded every 10 min over a 2-h period in the same conditions as used for plant growth. This measurement was made after 30 days of exposure to the different treatments, and water loss was expressed as percentage of the initial weight.

### Photosynthetic parameters

The gas exchange measurements were conducted in vivo on 30-d-old plants using an infrared gas analyzer (IRGA) LICOR-6400xt equipped with a fluorescence chamber. The IRGA was programmed to a photosynthetic active radiation (PAR) of 1000 µmol photons m^− 2^ s^− 1^, a temperature of 20 °C, a CO_2_ concentration of 400 µmol CO_2_ mol^− 1^, and 55–60% relative humidity. The measurements of net photosynthetic rate (P_n_), stomatal conductance (*g*_s_), and transpiration (*E*) were performed on the second leaflet of the third fully expanded leaf after light adaption for 2 h. Simultaneously, chlorophyll fluorescence was measured. The maximum quantum yield [F_v_´/F_m_´= (F_m_´-F_0_´)/F_m_´] was calculated according to [31], whereas the effective quantum yield of photosystem II [ΦPSII=(F_m_´-F_s_)/F_m_´)] and the absolute electron transport rate [ETR = ΦPSII*α(0.85)*β(0.5)*PPFD) were calculated according to [32].

### Determination of sugar, protein, and amino acid content

The metabolite measurements were made after 30 days of exposure to each treatment. Samples of fully expanded leaves were taken every 4 h (06:00, 10:00, 14:00, 18:00, and 22:00) during the light period, and were stored at -80 °C and subsequently lyophilized. The samples were extracted using 750 µL of pure methanol at 80 °C for 20 min. Afterwards, the samples were centrifuged at 165 *g* at 4 °C for 10 min. The supernatant was mixed with 375 µL of chloroform and 750 µL of ultra-pure water, centrifuged at 165 *g* at 4 °C for 10 min, and the upper phase was collected. Glucose, fructose, sucrose, and starch were determined enzymatically using spectrophotometry [33]. The total soluble protein concentration was determined by the Bradford method [34], using bovine serum albumin (BSA) as the standard. Total free amino acids were determined according to [35].

### Experimental design and statistical analyses

The experiments were set up in a randomized complete block design with six replicates. The data were tested using a one-way ANOVA and a Tukey test for the multiple comparisons. Correlation and Principal Component Analysis were used to establish relationships between the variables. All statistical analyses were undertaken using JMP Software 5.01® and a 5% level of significance.

## Results

### Electromagnetic spectrum and NP characterization

To corroborate the presence of UV-A light, we measured the electromagnetic spectrum inside the growth chamber. The first peak of the electromagnetic spectrum was indeed observed in the UV-A region (370 nm), while a second and third peak occurred in the visible, PAR region (400–700 nm) Fig. S1.

The NPs used in this work were confirmed as the rutile crystalline form by Raman spectrometry. They were rod-shaped, ranging in size from 30 to 60 nm (width) to 60–90 nm (length), with a hydrodynamic diameter of 236 nm, and a moderate tendency to agglomerate. For further details, see reference [21].

### Biomass production

**Fig. 1:**
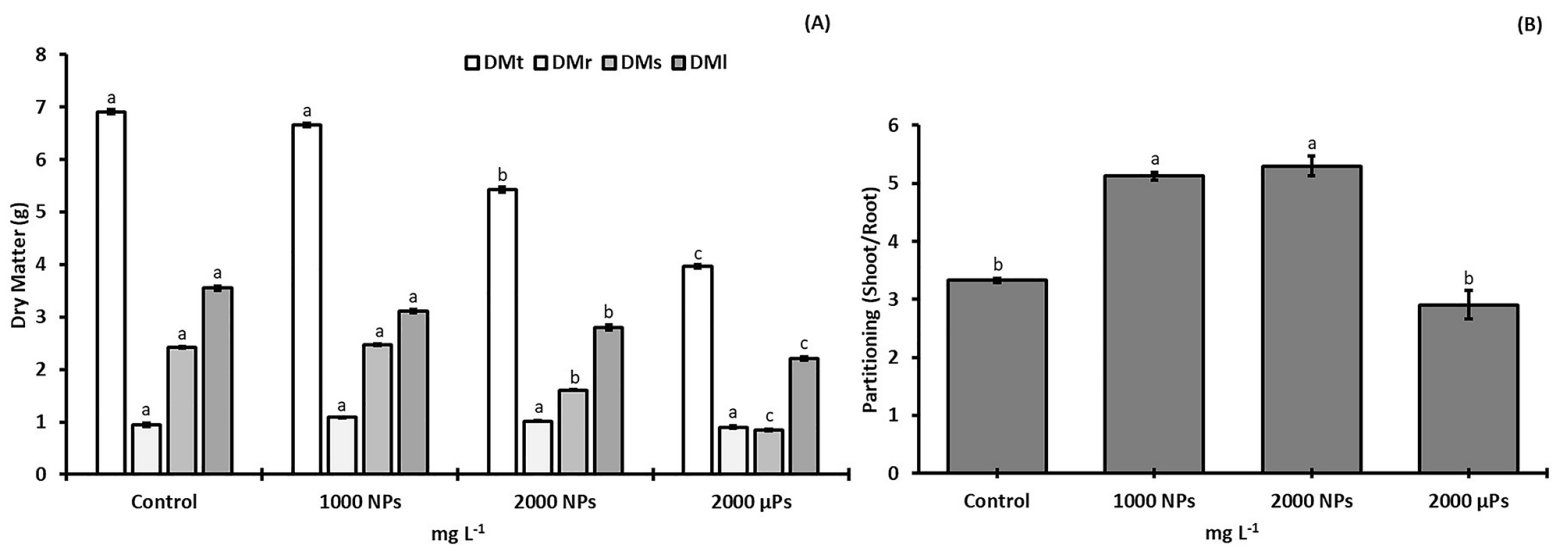
Biomass production of 30-d-old *S. lycopersicum* plants raised from seeds treated with TiO_2_ particles. (**A**) dry matter (DMt = total dry matter, DMr = root dry matter, DMs = stem dry matter, DMl = leaf dry matter), and (**B**) partitioning. For each parameter, different letters indicate significant differences using the Tukey test (*p* ≤ 0.05). Means ± SE (*n* = 5)

Tomato stem and leaf dry matter did not differ between the control and the 1000 mg NPs L^− 1^ treatments, but declined significantly at 2000 mg NPs L^− 1^ and even more so at the higher particle size (2000 mg µPs L^− 1^; Fig. 1A). No differences between treatments were noted in the root dry weight. The shoot/root ratio was significantly higher at 1000 and 2000 mg NPs L^− 1^ compared with the control and 2000 mg µPs L^− 1^ (Fig. 1B).

### Photosynthetic parameters

**Fig. 2:**
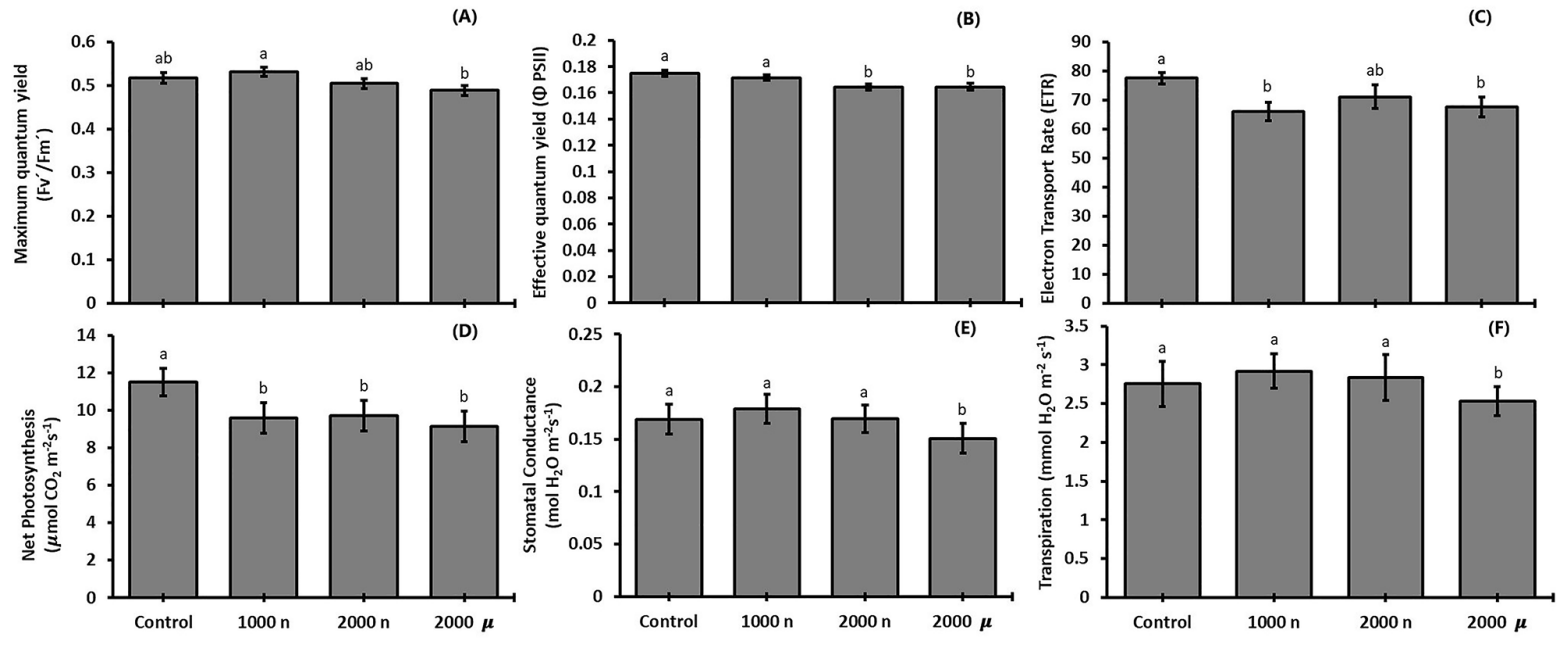
Photosynthetic performance of 30-d-old *S. lycopersicum* plants raised from seeds treated with TiO_2_ particles. (**A**) maximum light-adapted quantum yield, (**B**) effective quantum yield, (**C**) electron transport rate, (**D**) net photosynthetic rate, (**E**) stomatal conductance, and (**F**) transpiration. Different letters indicate significant differences using the Tukey test (*p* ≤ 0.05). Means ± SE, *n* = 5

As fluorescence and gas exchange parameters are two groups of relevant physiological indicators of plant photosynthetic performance, we measured them to determine whether NPs interact with the photosynthetic machinery. The parameters of chlorophyll fluorescence showed a decreasing trend as TiO_2_ concentration increased. The treatment with the higher particle size (2000 mg µPs L^− 1^) resulted in an 8% and 5.6% reduction in the maximum quantum yield of light-adapted (F_v_´/F_m_´) PSII compared with 1000 mg NPs L^− 1^, and control plants, respectively (Fig. 2A). The effective quantum yield (ΦPSII) of PSII and the Electron Transport Rate (ETR) were around 6% and 13% lower, respectively, in the higher dose and particle size (2000 mg µPs L^− 1^) than the control (Fig. 2B and C).

Related to the gas exchange parameters, net photosynthesis (P_n_) was highest (*p* ≤ 0.05) in the control and similar in the other treatments (Fig. 2D). For stomatal conductance (*g*_s_) and transpiration rate (*E*), reductions (*p* ≤ 0.05) of, 11% and 8% respectively occurred in the 2000 mg µPs L^− 1^ treatment compared with the control (Fig. 2E and F).

### Water loss measurement in cut leaves

**Fig. 3:**
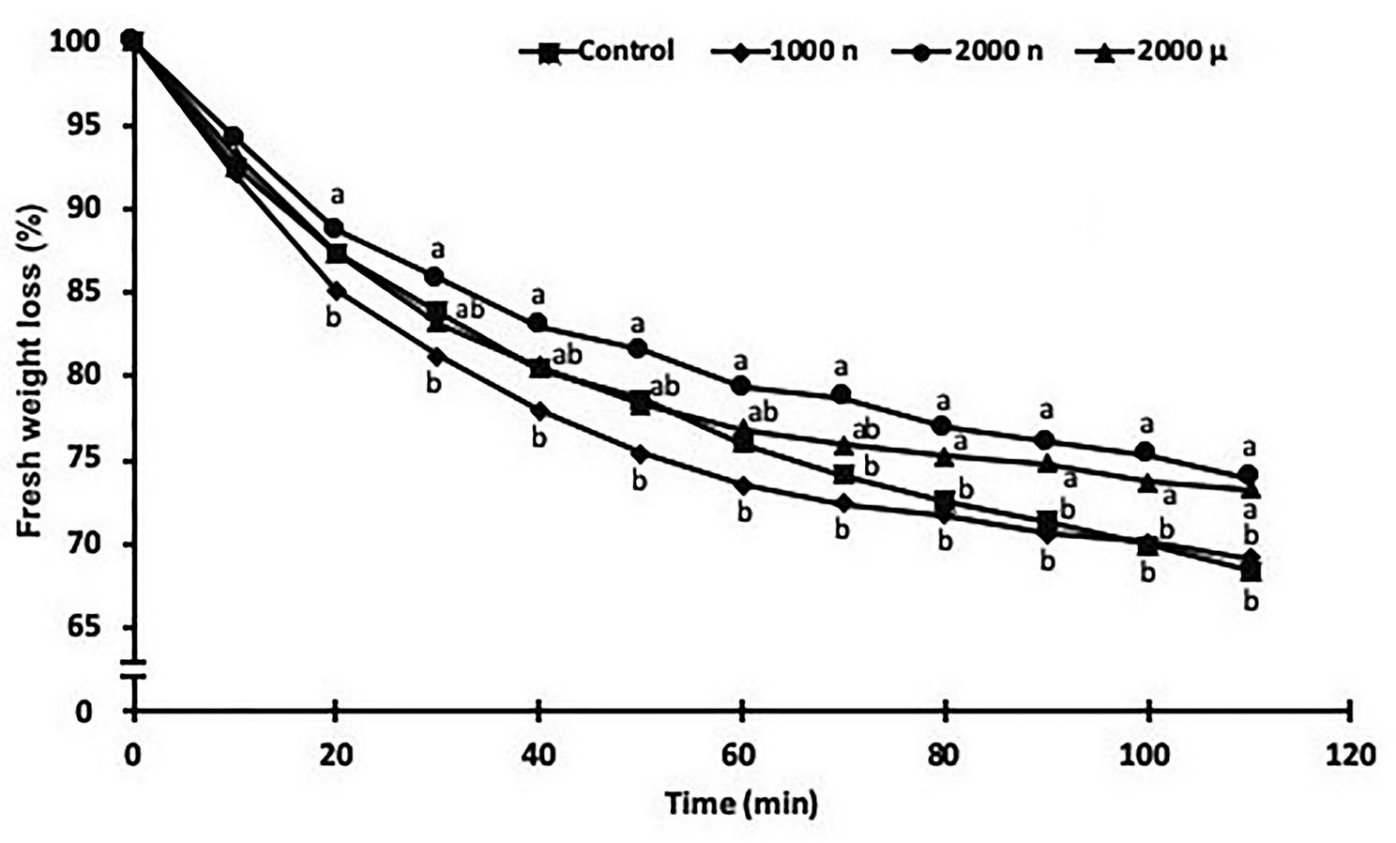
Water loss in leaves of 30-d-old *S. lycopersicum* plants grown from seeds treated with TiO_2_ particles. For each measurement time, different letters indicate significant differences between the treatments using the Tukey test (*p* ≤ 0.05). Means ± SE, *n* = 5

The assessment of water loss distinguished two stages, from 0 to 60 min and between 60 and 110 min (Fig. 3). In the first stage (0–60 min), water loss in tomato leaves treated with 1000 mg NPs L^− 1^ was significantly lower than in the 2000 mg NPs L^− 1^ treatment. Intermediate values were observed for control and 2000 mg µPs L^− 1^ conditions with no differences between them. In the second stage (60–110 min), the 1000 mg NPs L^− 1^ treatment and the control were similar, in that both suffered significantly higher water loss after 110 min than the 2000 mg NPs L^− 1^ (by around 32%) and 2000 mg µPs L^− 1^ treatments (by around 27%).

### Diurnal changes in the levels of carbohydrates

**Fig. 4:**
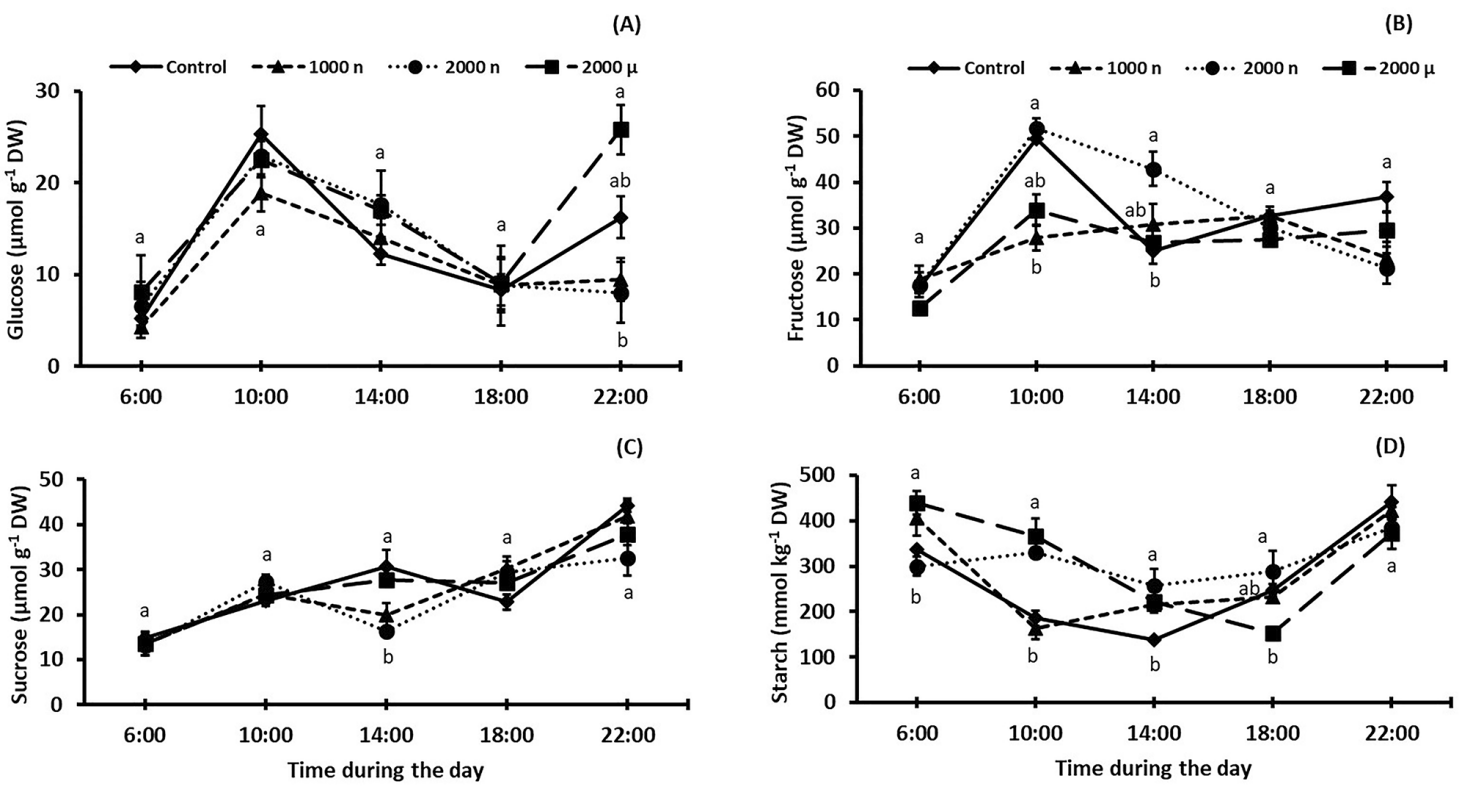
Carbohydrate concentration changes during daylight in leaves of 30-d-old *S. lycopersicum* plants grown from seeds treated with TiO_2_ particles. (**A**) glucose, (**B**) fructose, (**C**) sucrose, and (**D**) starch. For each measurement time, different letters indicate significant differences between the treatments using the Tukey test (*p* ≤ 0.05). Means ± SE, *n* = 5

The measurements of the daily variation in carbohydrate concentrations in leaves (Fig. 4) indicated a similar pattern for glucose for all treatments, except that at the end of the day, 2000 mg µPs L^− 1^ treated plants had more of this monosaccharide than 1000 mg NPs L^− 1^ and 2000 mg NPs L^− 1^ plants, with values similar to the control (Fig. 4A). The fructose concentration increased from 6:00 to 10:00 h in the control and under 2000 mg NPs L^− 1^ conditions and then decreased at 14:00 (more steeply in the control). There were no significant differences in fructose concentration at 18:00 and 22:00 (Fig. 4B). The sucrose concentration showed an increasing trend in all treatments throughout the light period, except at 14:00 when 1000 and 2000 mg NPs L^− 1^ treated plants had less than the control and 2000 mg µPs L^− 1^ tomatoes (Fig. 4C). The starch dynamics during the light period were similar for all treatments, starting with values that were higher (*p* ≤ 0.05) in 1000 mg NPs L^− 1^ and 2000 mg µPs L^− 1^ than in the control and 2000 mg NPs L^− 1^. Subsequently, a decrease followed by an increase occurred, with no significant differences between the treatments at the end of the light period (Fig. 4C).

### Diurnal changes in the protein and amino acid concentrations

**Fig. 5:**
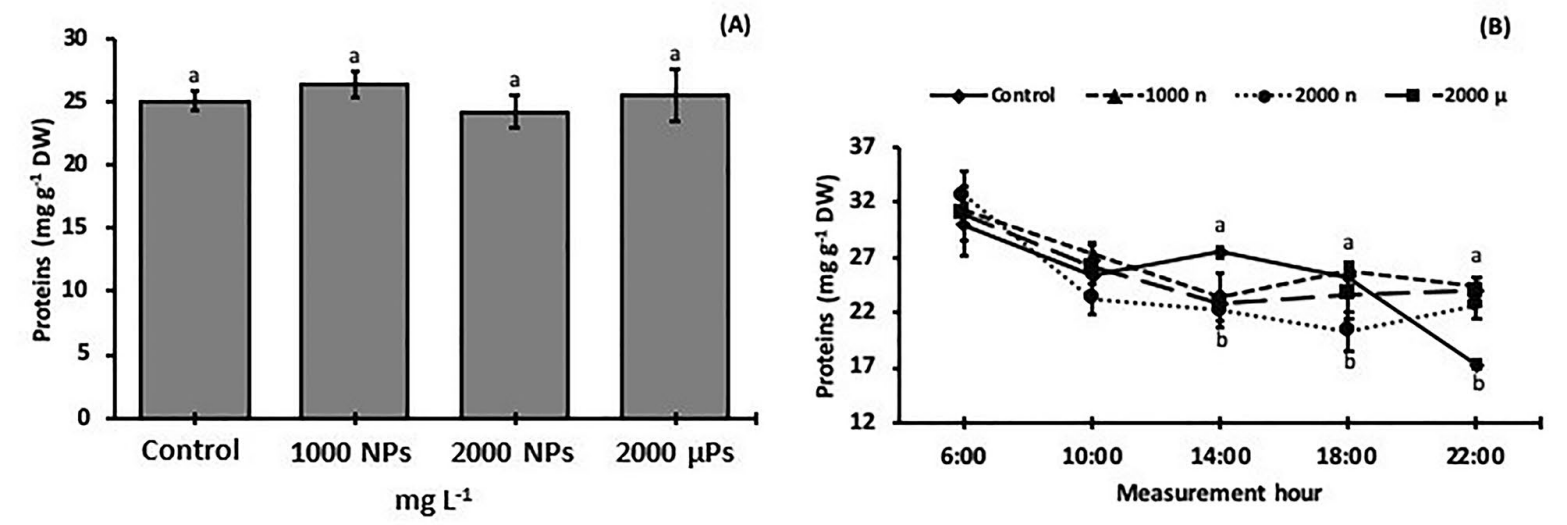
Total soluble proteins in leaves of 30-d-old *S. lycopersicum* plants grown from seeds treated with TiO_2_ particles. (**A**) average protein concentration at day 30, and (**B**) changes in protein concentration in leaves during the day. Different letters indicate significant differences between treatments (A) or at each measurement time (B) using the Tukey test (*p* ≤ 0.05). Means ± SE, *n* = 5

**Fig. 6:**
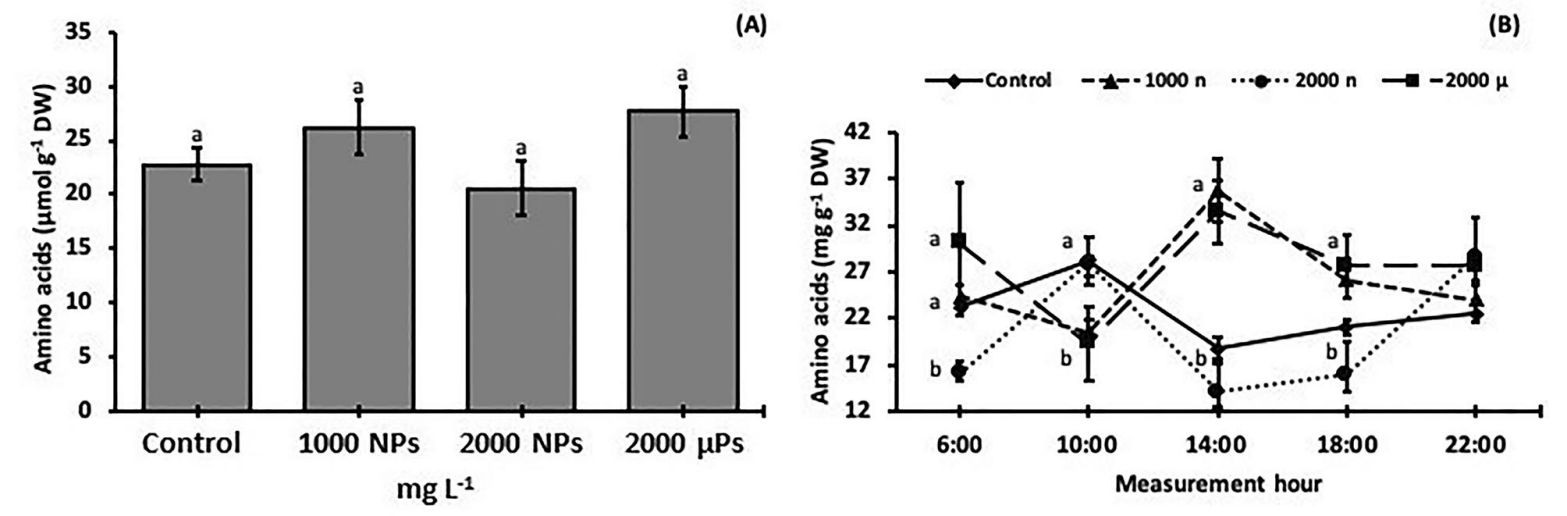
Amino acid concentrations in leaves of 30-d-old *S. lycopersicum* plants grown from seeds treated with TiO_2_ particles. (**A**) amino acid concentration and (**B**) amino acids concentration dynamics during day. Different letters indicate significant differences between treatments using the Tukey test (*p* ≤ 0.05). Means ± SE, *n* = 5

The total soluble protein levels in leaves of 30-d-old plants were similar among the treatments (Fig. 5A). Regarding the dynamics during the light period (Fig. 6B), 2000 mg NPs L^− 1^ treatments had less compared to the control from 14:00 to 18:00 h, and less than all the other treatments at the end of the light period (Fig. 5B).

The total amino acid concentrations were similar among the treatments (Fig. 6A). The dynamics during the light period (Fig. 6B) were similar in the control and 2000 mg NPs L^− 1^ between 10:00 to 18:00 h, characterized by higher values at the beginning, and lower values from the middle towards the end of the light period. The remaining two treatments (1000 mg NPs L^− 1^ and 2000 mg µPs L^− 1^) also had similar dynamics from 6:00 to 18:00 h, with values at 6:00 h and from 14:00 to 18:00 h higher than those at 10:00 h.

### Main relationships among the measured variables

**Fig. 7:**
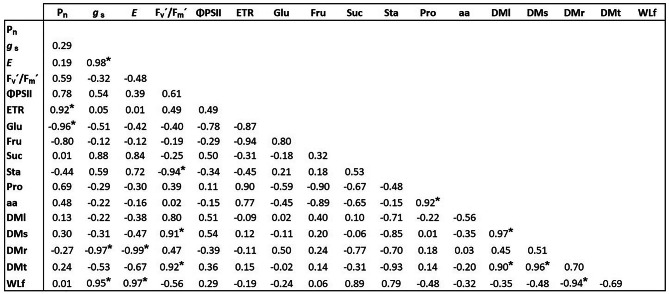
Pearson´s correlation matrix. * correlation significant at *p* ≤ 0.05. Abbreviations: P_n_ net photosynthetic rate; *E*, transpiration; *g*_s_, stomatal conductance; F_v_´/F_m_´, maximum quantum yield; ETR, electron transport rate; ΦPSII, effective quantum yield; Glu, glucose; Fru, fructose; Suc, sucrose; Sta, starch; Pro, proteins; aa, amino acids; WLf, water loss after 110 min; DMl, leaf dry matter; DMs, stem dry matter; DMr, root dry matter; DMt, total dry matter

Nineteen significant correlations were observed (four negative and 15 positive) (Fig. 7). The negative correlations were between starch and leaf, stem, and total dry matter. The positive correlations were associated mostly with gas exchange variables (*E* with *g*_s_), fluorescence (F_v_´/F_m_´ with ΦPSII), and gas exchange variables with fluorescence (*P*_n_ with ETR). In addition, ΦPSII was correlated negatively with starch and positively with leaf, stem, and total dry matter.

**Fig. 8:**
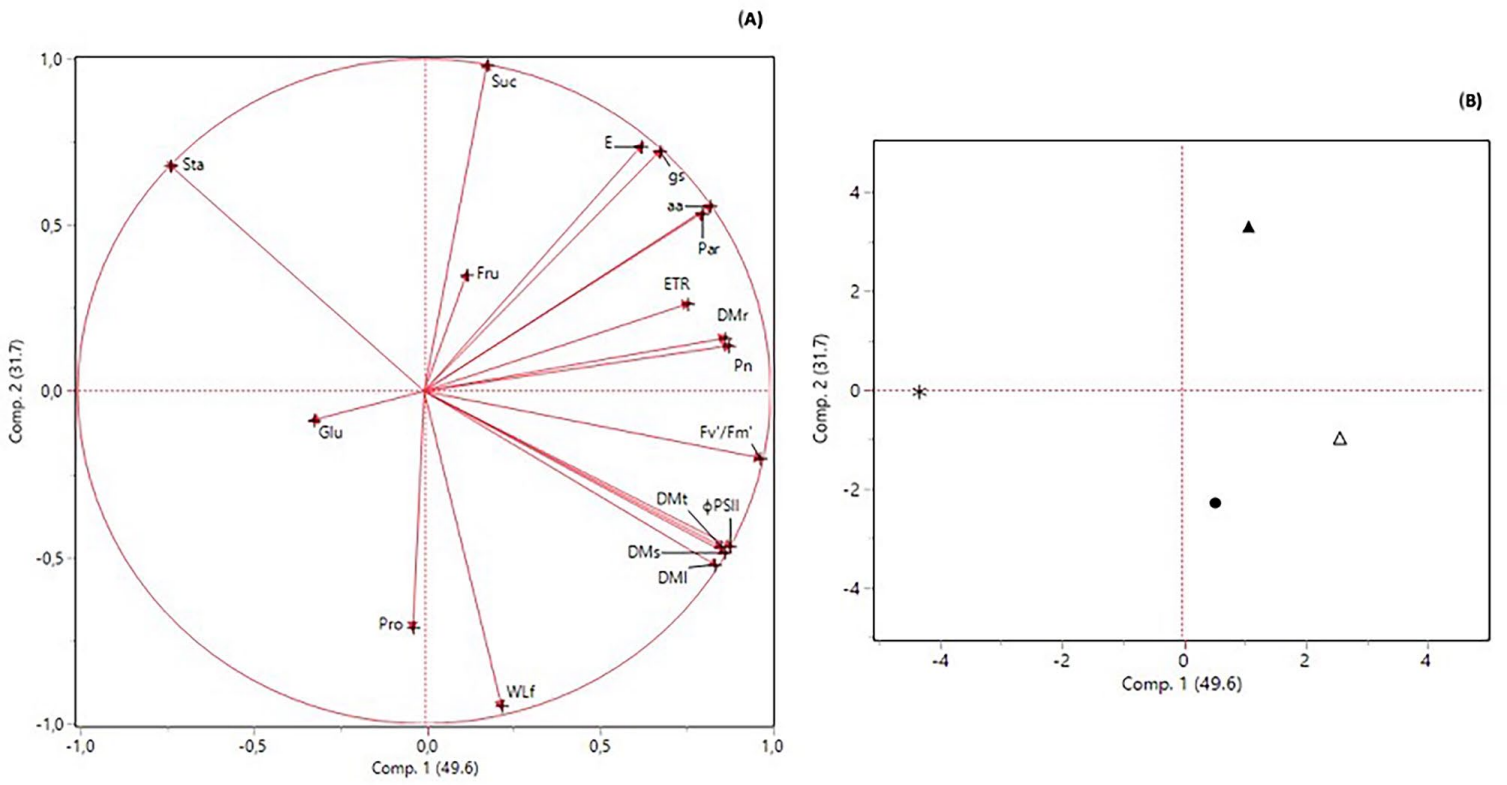
Principal Component Analysis (PCA) for all variables measured. (A) factorial charges plot and (B) score plot. In the score plot, the symbols represent control (●), 1000 mg NPs L^− 1^ (△), 2000 mg NPs L^− 1^ (▲), and 2000 mg µPs L^− 1^ (✽). Abbreviations: P_n_ net photosynthetic rate; *E*, transpiration; *g*_s_, stomatal conductance; F_v_´/F_m_´, maximum quantum yield; ETR, electron transport rate; ΦPSII, effective quantum yield; Glu, glucose; Fru, fructose; Suc, sucrose; Sta, starch; Pro, proteins; aa, amino acids; WLf, water loss after 110 min; DMl, leaf dry matter; DMs, stem dry matter; DMr, root dry matter; DMt, total dry matter

The spatial relations among variables are shown in Fig. 7. The first component of PCA explained 49.6% of the variance and included P_n_, F_v_´/F_m_´ ΦPSII, ETR, Glu, amino acids, and dry matter variables. The second component explained 31.7% of the variance and featured variables of gas exchange (*E* and *g*_s_), sugars, proteins, and total water loss after 110 min (Fig. 8A). The score plot (Fig. 8B) showed that 2000 mg NPs L^− 1^ (in quadrant I) were related to most variables. In quadrants II and III, only 2000 mg µPs L^− 1^ was present and associated with starch, glucose, and proteins. In quadrant IV the control and 1000 mg NPs L^− 1^ were associated with total water loss after 110 min and dry matter as well as some fluorescence parameters.

## Discussion

### Electromagnetic spectrum

The electromagnetic spectrum under which *S. lycopersicum* plants were grown ranged from 315 to 800 nm, with a UV-A peak at 370 nm (Fig. S1). This peak is appropriate for studying the photocatalytic properties of TiO_2_ NPs since in other studies, similar wavelengths were reported (392 nm) for the peak absorption of chemically synthesized rutile TiO_2_ [36]. Furthermore, other studies on photocatalysis have used UV-A peaks at 365 and 352 nm [37, 38].

### Titanium dioxide particles decrease gas exchange and chlorophyll fluorescence

The NP-plant interactions are species-specific and variable depending on the characteristics and concentration of the NPs, the developmental stage of the plant, and the means of exposure [foliar, root (soil amendments or nutrition solution), and seed imbibition] [20, 23, 39, 40]. Some authors have pointed out that TiO_2_ did not provoke acute toxicity in *S. lycopersicum* when applied at 260 mg anatase/rutile per kg soil [16]. An increase in photosynthesis performance is the most common effect of TiO_2_ NPs in several crop plants, such as *Spinacia oleracea* with 25 mg L^− 1^ rutile applied to seed [41], *S. lycopersicum* with foliarly applied 100 mg L^− 1^ anatase [24], and *O. basilicum* with a soil application of 750 mg kg^− 1^ rutile [42]. However, our results show adverse effects on chlorophyll fluorescence and gas exchange parameters in *S. lycopersicum* (Fig. 2). It is important to note that the experiments using TiO_2_ NPs only produce effects when exposed to sunlight because TiO_2_ energy absorption is in the UV-A region [25], which was provided artificially in our study (Fig. S1). The specific range of wavelengths associated with the light source used here could have overstimulated TiO_2_, triggering an increase in the electron transport chain in the photochemical phase, as described by [41]. Similarly, in previous work, excess energy impaired PSII of *Raphanus sativus* L. using anatase in concentrations from 10 to 1500 mg L^− 1^ applied to leaves [15]. This excess energy could explain the possible damage to PSII and, subsequently, the significant decrease of P_n_ in all TiO_2_ particle treatments and *g*_s_ and *E* in µPs (Fig. 2).

A major effect of seed-applied 50 mg TiO_2_ µPs L^− 1^ (rutile and anatase) on biochemical parameters (oxidative response) in *V. faba* was reported [43]. Such findings can be explained by the agglomeration of NPs to such an extent that they appear to mimic µPs [21], and which also leads to higher entry of µPs in *R. sativus* [15]. This is in line with [7], in which it was observed that the effects of TiO_2_ are influenced by the size of the particles. The decrease in PSII quantum yield due to TiO_2_ NP exposure has been observed in other plant species, such as *T. aestivum* (5-150 mg L^− 1^ of rutile/anatase mix applied to seeds) [44], *Ulmus elongata* (400 mg L^− 1^ anatase) [44], and *S. lycopersicum* (2000–4000 mg L^− 1^ rutile, root-applied) [20].

The effect on photosynthesis performance could be due to a decrease in uptake of nutrients involved in metabolic processes [20], as iron (Fe) and sulfur (S) concentrations decreased in *S. lycopersicum* leaves in the presence of 2000 mg L^− 1^ TiO_2_ NPs rutile applied to the soil. Similar results were also reported, indicating a decrease in S concentration in the leaf tissue of *S. lycopersicum* when treated with 1000 mg L^− 1^ TiO_2_ NPs applied to soil [45]. Iron is an essential micronutrient involved in photosynthesis, with about 80% of cellular Fe in the chloroplasts, where it functions as a redox-active metal [46]. In addition, Fe-S proteins (e.g., ferredoxin-thioredoxin system) are involved in electron transfer as part of substrate-binding enzyme sites [46, 47]. In plants with high Ti content, this element has been shown to compete with Fe for ligands or proteins [48]. Based on these findings, we hypothesize that lower PSII performance and possible Fe/S deficiency or lack of Fe-S proteins could have been induced by TiO_2_ treatments.

### Titanium dioxide NPs affect S. Lycopersicum biomass production

The higher biomass reduction that was observed in the 2000 mg µPs L^− 1^ treatment compared to the control and NP treatments could be related to the tendency of TiO_2_ NPs to agglomerate [21]. This reduction in biomass mainly affected stems and leaves and was therefore reflected in the root/shoot ratio (Fig. 1B). A similar study in the same species using 1000 mg NPs L^− 1^ applied to soil found a fall in leaf biomass but no changes in that of the root or stem [45]. Other authors used aerosol TiO_2_ NPs (anatase/rutile mix) on *S. lycopersicum* and found no changes in biomass production with concentrations from 50 to 5000 mg L^− 1^ [25]. However, some researchers have observed opposite results to our data, in that *S. lycopersicum* root and shoot biomass increased with respect to the control when 50 mg TiO_2_ NPs anatase per kg was applied to seed [49], a substantially lower concentration than we applied here. Thus, it appears that a fall in photosynthetic parameters provoke a decrease in biomass, in a manner that is dependent on concentration and particle size.

### Application of titanium dioxide particles does not affect leaf metabolites

TiO_2_ NPs (anatase) stimulated the synthesis of carbohydrates in leaves of species such as *U. elongata*, *T. aestivum*, and *S. lycopersic*um, whereby the concentrations of glucose, fructose and particularly sucrose increased with an increase in the concentration applied, probably due to a stress response to NPs [16, 22, 50, 51]. In the present study, we did not observe differences in the concentrations of these carbohydrates, probably due to the use of rutile as the crystalline form of TiO_2_ that is considered less toxic than anatase [7]. It should be pointed out that a mix of anatase and rutile TiO_2_ NPs at 260 mg mix per kg soil, gave no evidence of acute toxicity on *S. lycopersicum* [16].

Our data showed changes in sugar concentration during the day. In particular, at 1000 mg NPs L^− 1^, the glucose concentration was statistically higher at the end of the day, and that of starch was higher at the beginning of the day. This could be related to the increase in the storage of photosynthesis energy as starch, and a fall in the use of these reserves, as observed in *O. sativa* treated with TiO_2_ NPs anatase (root-applied) in concentrations from 0.1 to 100 mg L^− 1^ [52]. In the same study, an increase in the transformation of monosaccharides to disaccharides was observed, a finding not apparent in the present work. Indeed, treatment-induced changes in sugar concentrations during the day followed the typical cycle of production, exemplified by the continuous accumulation of sucrose, as well as an increase in starch from the middle of the day for use as sucrose at night [53].

We did not observe differences in the concentration of leaf proteins between the treatments, which is concordant with studies in *T. aestivum* using 250 to 2000 mg L^− 1^ of root-applied TiO_2_ NPs [54]. On the other hand, the total protein concentration increased in plants treated with TiO_2_ NPs anatase, such as in *S. oleracea* when 250 mg L^− 1^ were applied foliarly [55], *V. radiata* (L.) Wilczek at 10 mg L^− 1^ (foliarly) [56], and *O. sativa* at 100 to 500 mg L^− 1^ (root) [11]. In summary, we observed that reductions in photosynthetic rates by TiO_2_ treatments (Fig. 2D) did not impact the leaf concentrations of sugars (Fig. 4) and proteins (Fig. 5A).

## Conclusions

Titanium dioxide NPs applied to seeds of *S. lycopersicum* decreased the photochemical and gas exchange parameters, and consequently biomass, depending on their concentration and particle size. These negative effects did not impact sugar and protein concentrations in leaves. Further studies, such as determining the foliar concentration of macro and micronutrients, studying the dynamics of the activity of antioxidant enzymes and the content of low-molecular protective compounds, and examining leaf histology, are needed to elucidate the possible mechanisms that underlie the negative effects found in this study.

### Electronic supplementary material

Below is the link to the electronic supplementary material.

Supplementary Material 1

## Data Availability

The corresponding author has the datasets used during the current study and these data are available on reasonable request.
