# Supplementary figures and images for: Physiological and agronomical traits effects of titanium dioxide nanoparticles in seedlings of Solanum lycopersicum L
Source: BMC Plant Biol. 2024 Feb 28;24:146. doi: 10.1186/s12870-024-04763-9 (PMC10900795; doi:10.1186/s12870-024-04763-9)

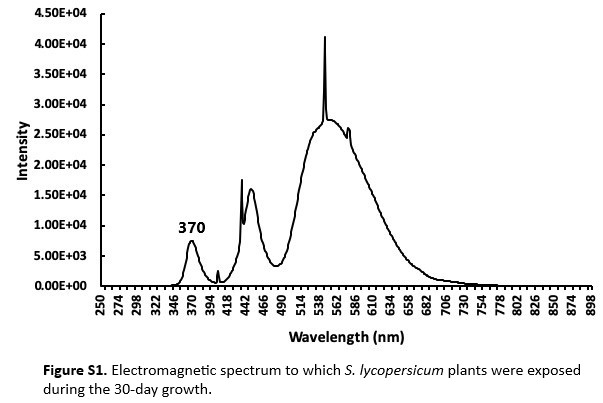

Supplement: Supplementary file 1 — Supplementary Material 1 [file 12870_2024_4763_MOESM1_ESM.jpg]
